# Zeiderholz's Self-Acting Blow-Pipe and Lamp

**Published:** 1850-10

**Authors:** 


*Zeiderholz's Self-acting Blow-pipe and Lamp.*—This lamp, although intended for druggists, assayists, chemists and apothecaries, as well as dentists, is admirably adapted for the laboratory of the latter. It furnishes a sufficient amount of heat for melting lead, tin, or zinc. It has two reservoirs for alcohol, and the flame from the surface of the one, which is the central, heats the alcohol in the other, which is a hollow cylinder surrounding this, causing rapid evaporation; the gas escaping through a tube that terminates in the center of the first reservoir, a little above the surface of the burning fluid, throws the flame against the bottom of the ladle placed a little above the lamp. We have had one in our laboratory for a little more than two months, and finding it very useful, we are induced to recommend it to the profession. It occupies but a little more room than an ordinary lamp, and may be obtained at the low price of five dollars, and we presume, in nearly all the large cities. We procured ours from Jones, White & Co.

